# Ponatinib Inhibits Multiple Signaling Pathways Involved in STAT3 Signaling and Attenuates Colorectal Tumor Growth

**DOI:** 10.3390/cancers10120526

**Published:** 2018-12-19

**Authors:** Fiona H. Tan, Tracy L. Putoczki, Jieqiong Lou, Elizabeth Hinde, Frédéric Hollande, Julie Giraud, Stanley S. Stylli, Lucia Paradiso, Hong-Jian Zhu, Oliver M. Sieber, Rodney B. Luwor

**Affiliations:** 1 Department of Surgery, The University of Melbourne, The Royal Melbourne Hospital, Parkville VIC 3050, Australia; Fiona.tan@petermac.org (F.H.T.); Putoczki.t@wehi.edu.au (T.L.P.); stanley.stylli@mh.org.au (S.S.S.); Lucialp@unimelb.edu.au (L.P.); Hongjian@unimelb.edu.au (H.-J.Z.); sieber.o@wehi.EDU.AU (O.M.S.); 2 Inflammation Division, The Walter and Eliza Hall Institute of Medical Research, Parkville VIC 3052, Australia; 3 Department of Medical Biology, The University of Melbourne, Parkville VIC 3050, Australia; 4 Department of Biochemistry and Molecular Biology, Bio21, The University of Melbourne, Parkville VIC 3010, Australia; jieqiong.lou@unimelb.edu.au (J.L.); Elizabeth.hinde@unimelb.edu.au (E.H.); 5 Department of Clinical Pathology, Victorian Comprehensive Cancer Centre, The University of Melbourne, Melbourne VIC 3000, Australia; frederic.hollande@unimelb.edu.au; 6 CNRS, Institut de Génomique Fonctionnelle, Universités de Montpellier 1 & 2, UMR-5203, INSERM U661, 34094 Montpellier, France; julie.giraud085@gmail.com; 7 Department of Neurosurgery, The Royal Melbourne Hospital, Parkville VIC 3050, Australia; 8 Systems Biology and Personalised Medicine Division, The Walter and Eliza Hall Institute of Medical Research, Parkville VIC 3052, Australia; 9 School of Biomedical Sciences, Monash University, Clayton VIC 3168, Australia

**Keywords:** STAT3, CRC, Ponatinib, EGFR, interleukin signaling

## Abstract

Signal transducer and activator of transcription 3 (STAT3) signaling is a major driver of colorectal cancer (CRC) growth, however therapeutics, which can effectively target this pathway, have so far remained elusive. Here, we performed an extensive screen for STAT3 inhibitors among a library of 1167 FDA-approved agents, identifying Ponatinib as a lead candidate. We found that Ponatinib inhibits STAT3 activity driven by EGF/EGFR, IL-6/IL-6R and IL-11/IL-11R, three major ligand/receptor systems involved in CRC development and progression. Ponatinib was able to inhibit CRC migration and tumor growth in vivo. In addition, Ponatinib displayed a greater ability to inhibit STAT3 activity and mediated superior anti-proliferative efficacy compared to five FDA approved SRC and Janus Kinase (JAK) inhibitors. Finally, long-term exposure of CRC cells to Ponatinib, Dasatinib and Bosutinib resulted in acquired resistance to Dasatinib and Bosutinib occurring within six weeks. However, acquired resistance to Ponatinib was observed after long-term exposure of >4 months. Overall, our results identify a novel anti-STAT3 property of Ponatinib and thus, Ponatinib offers a potential therapeutic strategy for CRC.

## 1. Introduction

Colorectal cancer (CRC) is one of the leading causes of cancer mortality [1,2]. First and second-line therapy in the metastatic setting consists of fluoropyrimidine based chemotherapy, in which 5-fluorouracil or capecitabine is combined with either oxaliplatin or irinotecan [3,4,5]. Targeted therapy has emerged as a treatment option for CRC patients. These include the epidermal growth factor receptor (EGFR) inhibitors cetuximab and panitumumab and the vascular endothelial growth factor an inhibitor bevacizumab [6,7,8]. However, despite these agents producing improvements in patient outcome, treatment failure is frequently observed and 5-year survival rates for patients with metastatic disease remains below 15% [9,10]. These disappointing outcomes highlight the requirement for continued evaluation for improved therapeutic agents, in particular those that target critical oncogenic molecules and pathways.

Signal transducer and activator of transcription 3 (STAT3) is a pro-tumorigenic transcription factor that is frequently hyper-activated in many types of tumors including CRC [11]. Considerable evidence has demonstrated an essential role for STAT3 in the regulation of genes such as SOCS3, cyclin D1 and HIF1α in promoting tumor cell proliferation, migration, invasion and resistance to therapies [11,12]. STAT3 is phosphorylated in response to the activation of several cytokine receptors in the IL-6 cytokine receptor family in conjunction with their co-receptor gp130 and by growth factor receptors including the EGFR [13]. Both IL-6 and EGFR play critical roles in the pro-oncogenic properties of STAT3 [14,15,16]. Recently, another IL-6 cytokine family member, IL-11 was observed in high abundance in CRC tumor samples and correlated with increased phosphorylated STAT3 levels [17]. In addition, non-receptor kinases including SRC and Janus Kinase (JAK) also activate STAT3 [18,19]. Therefore, targeting the STAT3 signaling axis represents an important and rationale approach for the clinical management of patients with CRC. 

However, direct inhibitors of STAT3 have not progressed beyond early-phase clinical trials [20,21]. Agents that target molecules upstream of STAT3 such as SRC and JAK inhibitors have also demonstrated modest clinical efficacy [22,23], which may at least in part be due to other uninhibited ligand–receptor systems inducing compensatory signaling and subsequently allow for the re-activation of critical tumor promoting downstream molecules such as STAT3. Indeed, we and others have demonstrated that blocking one receptor is not sufficient in inhibiting STAT3 activity, as other uninhibited pathways such as those driven by EGFR, IL-6R and IL-11R can reactivate STAT3, leading to continued tumor growth and refractory outcomes clinically [16,24,25]. Given that (1) STAT3 is consistently found to be hyper-activated in CRC; (2) STAT3 pro-tumorigenic properties are commonly mediated through EGF, IL-6 and IL-11 ligand/receptor systems in CRC; (3) STAT3 is often re-activated through uninhibited ligand/receptor systems; and (4) a successful anti-STAT3 agent has yet to be approved in any cancer setting, we explored the possibility of identifying agents currently approved for other indications that may also display novel anti-STAT3 activity. Importantly, identifying an agent that could inhibit STAT3 activity driven through an EGF, IL-6 and IL-11 ligand/receptor system may limit compensatory or re-activation of STAT3 and potentially reduce the likelihood of tumor resistance. 

One of the most successful tyrosine kinases inhibitors in cancer treatment is the BCR-ABL inhibitor Imatinib, an effective first-line therapy for patients with chronic myeloid leukemia (CML) [26,27,28]. However, resistance to Imatinib is commonly observed, leading researchers to generate second-line BCR-ABL inhibitors including Dasatinib [29] and Nilotinib [30]. Kinase domain mutations in BCR-ABL, particularly the BCR-ABL^T315I^ mutation, confer resistance to Dasatinib, Nilotinib and Imatinib. Therefore, a third generation agent, Ponatinib (Iclusig™; ARIAD Pharmaceuticals, Cambridge, MA, U.S.A.) [31,32] was designed as a pan-inhibitor of BCR-ABL including the BCR-ABL^T315I^ mutation and has shown exciting results in CML patients who harbor the BCR-ABL^T315I^ mutation [33,34].

Here we identify Ponatinib from a large panel of approved therapeutics, to have an additional novel property of inhibiting STAT3 activity driven by multiple ligand-receptor systems. Ponatinib displayed superior inhibition of STAT3 activity compared to currently approved SRC and JAK inhibitors. We also demonstrate that Ponatinib can successfully inhibit CRC cell migration and tumor growth in vivo. In summary, Ponatinib has the potential to be re-purposed as a STAT3 inhibitor for the treatment of patients with CRC.

## 2. Results

### 2.1. IL-11-STAT3 Signaling Enhances Tumor Growth

**Figure 1:**
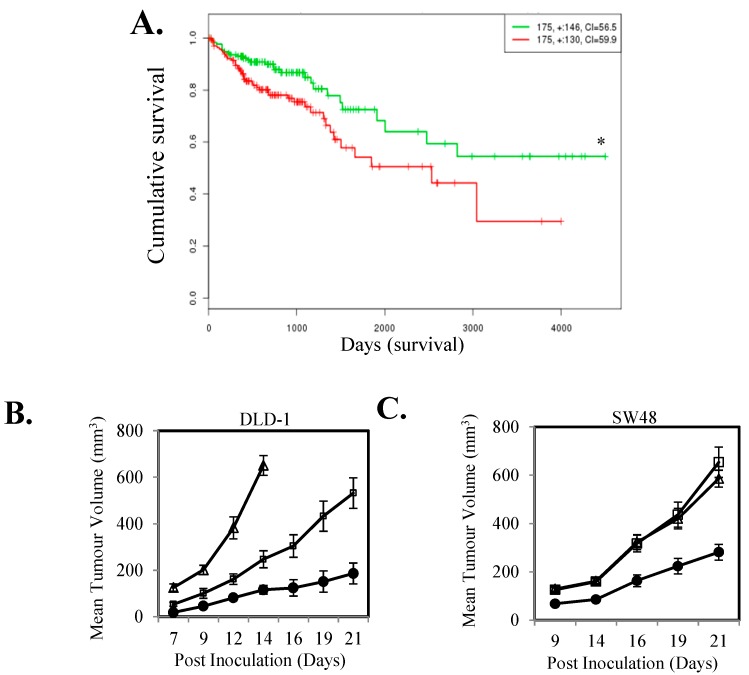
IL-11 signaling is associated with poorer survival in colorectal cancer (CRC) patients and drives increased tumor growth. (**A**) The relationship between high (Green) and low (Red) IL-11-IL-11Rα-SOCS3 gene expression with patient survival was determined through mining a SurvExpress TCGA dataset. Kaplan-Meier survival curves were evaluated from the TCGA, *n* = 350; Risk group hazard ratio = 1.13 (Conf. Int. 1.15~2.93); * *p* < 0.05. (**B**) DLD-1 control (●) and IL-11Rα transfected stable clones (IL-11R-1 (□) and IL-11R-2 (Δ)) and (**C**) SW48 control (●) and IL-11Rα transfected stable clones (IL-11R-1 (□) and IL-11R-2 (Δ)) were inoculated subcutaneously into both flanks of BALB/c^nu−/nu−^ female mice and measured for tumor volume. Data shown represents mean ± SEM (*n* = 10 tumors/group).

To investigate if a high level of IL-11-STAT3 signaling correlates with patient outcome in CRC, we examined if the association of expression levels of IL-11, IL-11R and SOCS3 correlated with outcome in the TCGA cohort. CRCs with high composite expression of these three readouts of STAT3 signaling had significantly worst overall survival compared to tumors with low composite expression (*n* = 350; Figure 1A). To directly determine the effect of activated STAT3 signaling on tumor growth we next stably transfected the IL-11Rα subunit into DLD-1 and SW48 CRC cells. IL-11Rα over-expressing cells displayed increased STAT3 activity and SOCS3 gene expression (Figure S1) and enhanced subcutaneous xenograft growth compared to parental cells (Figure 1B,C).

### 2.2. Ponatinib Inhibits STAT3 Phosphorylation and Transcriptional Activity

**Figure 2:**
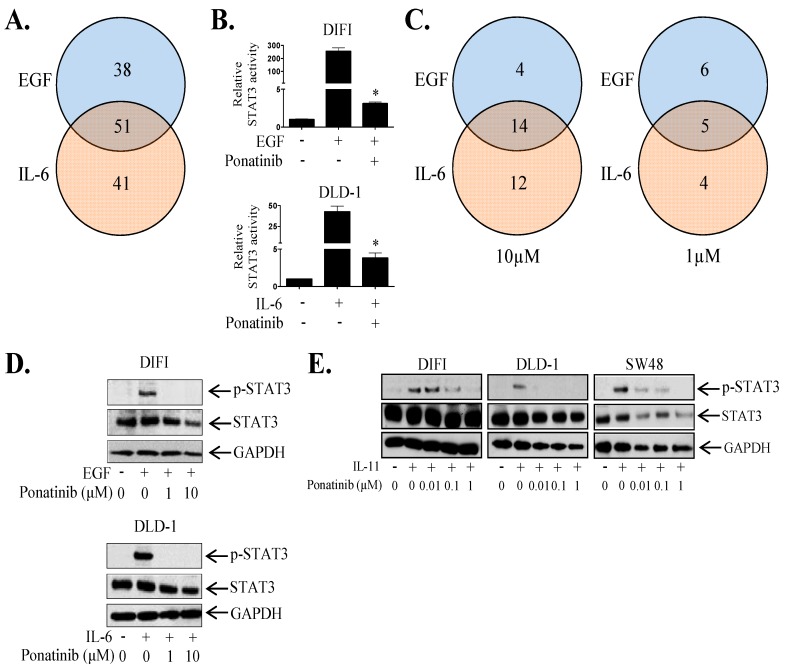
Ponatinib inhibits EGF, IL-6 and IL-11 mediated STAT3 activity. (**A**) Venn diagram summarizing the number of agents that could inhibit EGF, IL-6 or both EGF and IL-6 mediated STAT3 transcriptional activity by greater than 50% compared to control treated cells. (**B**) The effect of Ponatinib on EGF and IL-6 mediated STAT3 transcriptional activity. Data represents relative luciferase activity relative to control, mean ± SD, * *p* < 0.001. (**C**) Venn diagrams summarizing the number of agents that could inhibit EGF, IL-6 or both EGF and IL-6 mediated STAT3 phosphorylation by greater than 50% compared to control treated cells at 1 μM and 10 μM as determined by densitometry of western blot bands. Cells were treated with (**D**) EGF or IL-6 ± Ponatinib, or (**E**) IL-11 ± Ponatinib and then assessed for Phospho-STAT3, STAT3 and GAPDH expression by western blot.

It is clear that IL-11-STAT3 signaling plays a central role in cancer progression [17]. However, many receptor systems including the EGFR and IL6R activate STAT3, potentially leading to compensatory re-activation of STAT3 when only one of these receptor systems is inhibited. Thus, we set out to identify novel inhibitors that could block STAT3 activation driven by all three receptor systems (EGFR, IL-6R and IL-11R) amongst a panel of 1167 FDA-approved agents. We used a luciferase-based screen where DIFI and DLD-1 cells were infected with an STAT3 reporter adenovirus and then stimulated with either EGF (DIFI cells) or IL-6 (DLD-1 cells) with or without each inhibitor at 10 µM. The DIFI and DLD-1 cell lines were chosen based on their high STAT3 transcriptional activity in response to EGF and IL-6 respectively. Our initial screen identified 89 and 92 agents that could inhibit EGF or IL-6 mediated STAT3 transcriptional activity respectively by at least 50% at 10 µM (Figure 2A; Table S1). Fifty-one of these agents could inhibit both EGF (in DIFI cells) and IL-6 (in DLD-1 cells) driven STAT3 activity (Figure 2A,B; Table S2). A secondary screen of these 51 agents identified 18 and 26 agents that could inhibit EGF or IL-6 mediated STAT3 phosphorylation respectively by at least 50% at 10 µM (Figure 2C; Table S2). Fourteen of these agents could inhibit both EGF (in DIFI cells) and IL-6 (in DLD-1 cells) driven STAT3 phosphorylation (Figure 2C,D; Table S2). Amongst these inhibitors, 11 and 9 agents inhibited EGF or IL-6 mediated STAT3 phosphorylation at the lower dose of 1 μM, respectively (Figure 2C; Table S2). Five of these agents (including Ponatinib) reduced both EGF and IL-6 induced STAT3 phosphorylation at 1μM (Figure 2C,D; Table S2). Ponatinib also inhibited EGF and IL-6 induced SOCS3 gene expression (Figure S2).

As IL-11 clearly enhances CRC tumorigenicity, we concurrently evaluated whether the 51 agents that could inhibit EGF and IL-6 mediated STAT3 activity, could also block IL-11 driven STAT3 activation. Thirteen out of 51 agents reduced IL-11 mediated STAT3 transcriptional activity by greater than 50% at 1 µM in DLD-1, SW48 and LIM1215 cells (Table S2). Of these 13, only Ponatinib demonstrated the ability to inhibit STAT3 phosphorylation and transcriptional activity driven by all three ligands in all cell lines tested across our screening process. Ponatinib reduced IL-11 mediated STAT3 phosphorylation (Figure 2E, Figure S3A). Similarly to IL-6 and IL-11, Ponatinib reduced STAT3 phosphorylation induced by another IL-6 cytokine family member, LIF in a dose dependent manner in DIFI, DLD-1 and SW48 (Figure S3D).

### 2.3. Reduces STAT3 Localization and Function in the Nucleus

**Figure 3:**
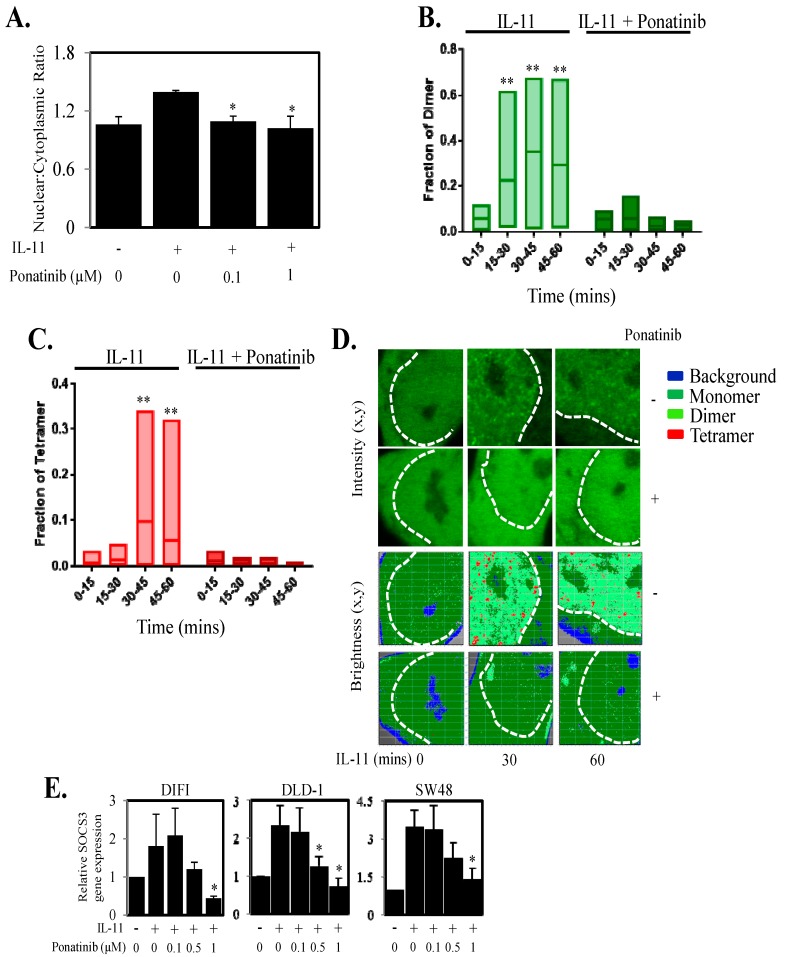
Ponatinib Inhibits STAT3 nuclear localization, dimer and tetramer formation and STAT3 regulated gene expression. (**A**) DLD-1 cells were transfected with STAT3-GFP for 48 h, and then treated with IL-11 with or without 0.1 µM and 1.0 µM Ponatinib for 1 h. Cells were then fixed, permeabilized and stained with DAPI. Nuclear and cytoplasmic localization of STAT3 was then performed and presented as a ratio of nuclear compared to cytoplasmic localization * *p* < 0.05 relative to IL-11 stimulated cells (*n* = 3). DLD-1 cells were transfected with STAT3-GFP and assessed for (**B**) STAT3 dimer and (**C**) tetramer formation upon stimulation with IL-11 ± Ponatinib (1 µM) (*n* = 8 cells). (**D**) Intensity and brightness maps of STAT3-GFP oligomerization at 0, 30 and 60 min after IL-11 stimulation ± Ponatinib in DLD-1 cells. (**E**) Cells were treated with IL-11 ± Ponatinib and then assessed for SOCS3 gene expression by qPCR; * *p* < 0.05; ** *p* < 0.01 relative to control.

As Ponatinib reduced STAT3 phosphorylation and transcriptional luciferase activity, we next explored whether Ponatinib could inhibit STAT3 localization and transcriptional function in the nucleus. DLD-1 cells transiently transfected with STAT3-GFP and stimulated with IL-11 displayed an increase in STAT3 nuclear to cytoplasmic ratio compared to unstimulated cells (1.39 vs. 1.06) (Figure 3A). Ponatinib significantly reduced the STAT3 nuclear to cytoplasmic ratio mediated by IL-11 at both 0.1 µM (1.09) and 1 µM (1.02) (Figure 3A). As STAT3 dimer and tetramer formation in the nucleus has been shown to be important for regulating gene expression [35] we next determined if Ponatinib could reduce STAT3 dimer and tetramer formation. DLD-1 cells transiently transfected with STAT3-GFP and stimulated with IL-11 displayed an increase in STAT3 dimer and tetramer formation within 15–30 min of stimulation (Figure 3B,C), while Ponatinib significantly inhibited IL-11 induced STAT3 dimer and tetramer formation in DLD-1 cells equivalent to basal levels (Figure 3B–D). Finally, as Ponatinib reduced STAT3 nuclear localization and dimer and tetramer formation in the nucleus we examined if Ponatinib could reduce gene expression driven by STAT3. IL-11 triggered increased SOCS3 gene expression in DIFI, DLD-1 and SW48 cells while Ponatinib reduced this IL-11 mediated SOCS3 gene expression in a dose dependent manner (Figure 3E). This was further confirmed in four other CRC cell lines (Figure S2C).

### 2.4. Ponatinib Displays a Broader Range of Anti-STAT3 Activity Compared to SRC and JAK Inhibitors

**Figure 4:**
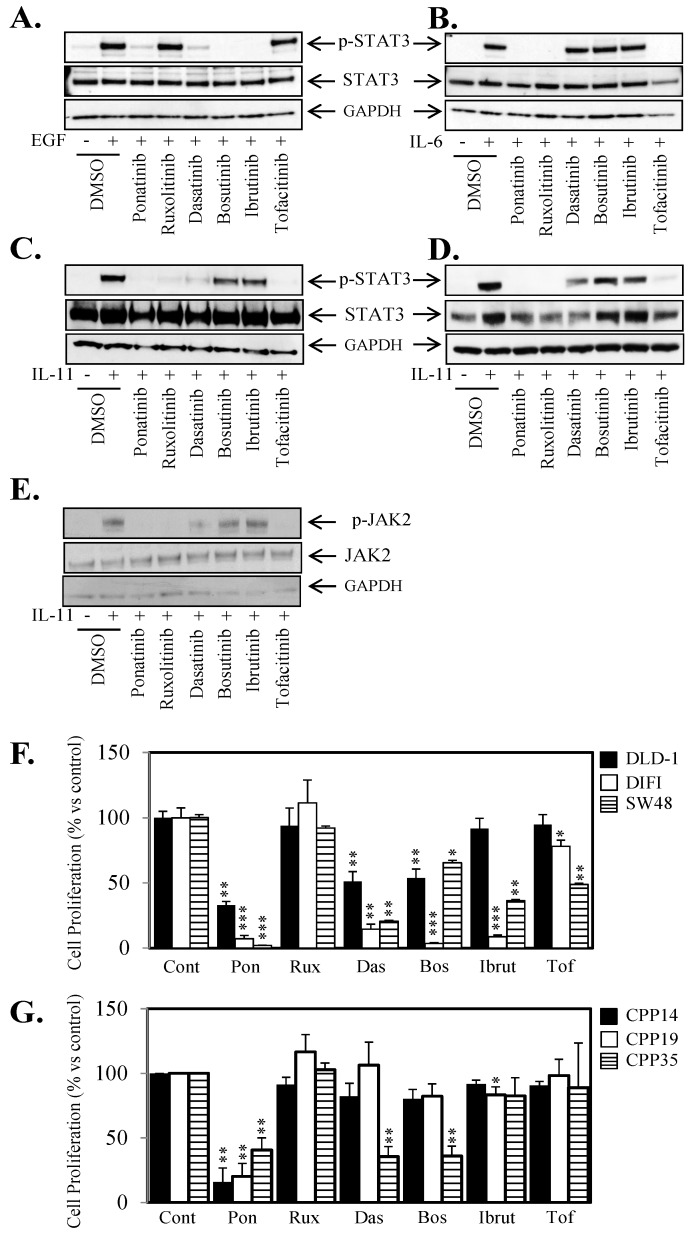
Ponatinib displays broader STAT3 inhibition compared to SRC and JAK inhibitors. Cells were treated with (**A**) EGF, 50 ng/mL (DIFI), (**B**) IL-6, 50 ng/mL (DLD-1), (**C**) IL-11, 100 ng/L (DLD-1), (**D**) IL-11, 100 ng/L (SW48) ± 1 µM Ponatinib, Ruxolitinib, Dasatinib, Bosutinib, Ibrutinib or Tofacitinib for 1 h and then assessed for Phospho-STAT3, STAT3 and GAPDH expression by western blot. (**E**) DLD-1 cells were treated with IL-11, 100 ng/mL ± 1 µM Ponatinib, Ruxolitinib, Dasatinib, Bosutinib, Ibrutinib or Tofacitinib for 1 h and then assessed for Phospho-JAK2, JAK2 and GAPDH expression by western blot. (**F**) DLD-1 (■), DIFI (□) and SW48 (horizontal lines) and (**G**) CPP14 (■), CPP19 (□) and CPP35 (horizontal lines) were treated with ± 1 µM Ponatinib (Pon), Ruxolitinib (Rux), Dasatinib (Das), Bosutinib (Bos), Ibrutinib (Ibrut) or Tofacitinib (Tof) for 72 h. Cell viability was then determined using a commercially available Cell Titer-Glo kit and samples read on a bioluminometer. Data is expressed as % viability compared to untreated control cells ± S.D of at least 3 independent experiments, each with 3 experimental replicates; * *p* < 0.05; ** *p* < 0.01; *** *p* < 0.001.

We next sought to investigate how Ponatinib’s additional ability to inhibit STAT3 activity compared to five other FDA approved agents (Ruxolitinib, Dasatinib, Bosutinib, Ibrutinib and Tofacitinib) which inhibit amongst other targets the SRC or JAK kinases directly upstream of STAT3. Ponatinib was the only agent that could significantly inhibit STAT3 activity driven by EGF (Figure 4A, Figure S4A), IL-6 (Figure 4B, Figure S4B), and IL-11 (Figure 4C,D, Figure S4C,D). Unlike Ponatinib, the inhibitors that could block SRC and JAK activity could only inhibit STAT3 activity driven by one or two of the ligand/receptor systems tested but not all three. We also demonstrated that Ponatinib was able to inhibit IL-11-mediated JAK2 phosphorylation (Figure 4E). Interestingly, the inhibitors that could block IL-11 mediated STAT3 activity (Ponatinib, Ruxolitinib and Tofacitinib) also blocked IL-11-mediated JAK2 activity, and those that could not (Dasatinib, Bosutinib, Ibrutinib) also did not block JAK2 activity. Similarly, Ponatinib was the only agent that could inhibit cell viability in all seven colon cancer cell lines and three primary CRC cell lines (Figure 4F,G; Figure S4E) by greater than 50%.

**Figure 5:**
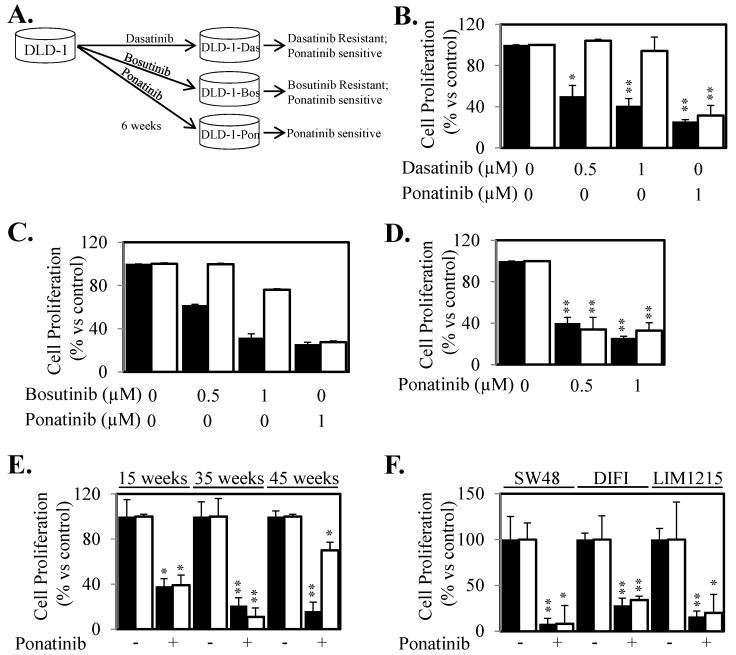
Cells do not acquire resistance to Ponatinib as rapidly as Dasatinib and Bosutinib. (**A**) Schematic of long-term treatment with Dasatinib, Bosutinib and Ponatinib. DLD-1 cells were treated with continuous, increasing doses of Dasatinib, Bosutinib and Ponatinib for six weeks and then assessed for cell viability in comparison to control DLD-1 cells. (**B**) DLD-1 (■) and DLD-1-Das cells (□), were treated with ± Dasatinib or Ponatinib for 72 h. Cell viability was then determined using a commercially available Cell Titer-Glo kit and samples read on a bioluminometer. Data is expressed as % viability compared to untreated control cells ± S.D. (**C**) DLD-1 (■) and DLD-1-Bos cells (□), were treated with ± Bosutinib or Ponatinib for 72 h. Cell viability was then determined as outlined above. (**D**) DLD-1 (■) and DLD-1-Pon cells (□), were treated with ± Ponatinib for 72 h. Cell viability was then determined as outlined above. (**E**) DLD-1 (■) and DLD-1 cells that had been co-cultured in the presence of Ponatinib for 15, 35 and 45 weeks (□), were treated with ± Ponatinib for 72 h. Cell viability was then determined as outlined above. (**F**) SW48, DIFI and LIM1215 cells (■) and their counterparts that had been co-cultured in the presence of Ponatinib for greater than four months (□), were treated with ± Ponatinib for 72 h. Cell viability was then determined as outlined above from at least three independent experiments, each with 3 experimental replicates; * *p* < 0.05; ** *p* < 0.01.

Continued or re-activated compensatory downstream signaling initiated by alternative un-inhibited receptors often provides acquire resistance to current targeted therapeutics. Our present data led us to speculate that Ponatinib may have superior properties in preventing re-activation of STAT3 compared to other inhibitors that can block SRC and JAK activity due to its ability to inhibit three key receptors involved in STAT3 activation. To evaluate the potential of acquired resistance to either Ponatinib or the SRC and JAK inhibitors we co-cultured DLD-1 cells with continuous, increasing doses of Dasatinib, Bosutinib and Ponatinib for six weeks (Figure 5A). Cells cultured in the presence of Ponatinib did not proliferate when doses were increased and thus a maintenance dose of 0.1 μM was used throughout the six weeks treatment period. Cells treated with Dasatinib or Bosutinib however could tolerate small incremental dose increases over the six weeks treatment period until a final dose of 1.5 μM was reached. Strikingly, cells treated long-term with Dasatinib (designated DLD-1-Das) or Bosutinib (designated DLD-1-Bos) displayed enhanced resistance within six weeks of treatment but importantly, these Dasatinib-refractory and Bosutinib-refractory cells maintained their sensitivity to Ponatinib at comparable levels to that of the parental cell line (Figure 5B,C). However, cells that were co-cultured in the presence of Ponatinib (designated DLD-1-Pon) remained equally as sensitive to Ponatinib as the Ponatinib-treatment naïve parental DLD-1 cells (Figure 5D).

To determine whether acquired resistance to Ponatinib would arise from longer continuous treatment, we cultured DLD-1, SW48, DIFI and LIM1215 cells in the continuous presence of Ponatinib for several months. DLD-1 cells treated with Ponatinib for 15 and 35 weeks continued to demonstrate similar sensitivity to Ponatinib compared to untreated parental DLD-1 cells (Figure 5E). Acquired resistance was finally observed after 45 weeks of Ponatinib exposure (Figure 5E). Similarly, SW48, DIFI and LIM1215 cells continuously cultured in the presence of Ponatinib for at least 20 weeks maintained a similar sensitivity to Ponatinib compared to treatment-naïve parental cells (Figure 5F). 

### 2.5. Ponatinib Inhibits Cell Proliferation, Migration and Tumor Growth In Vivo

**Figure 6:**
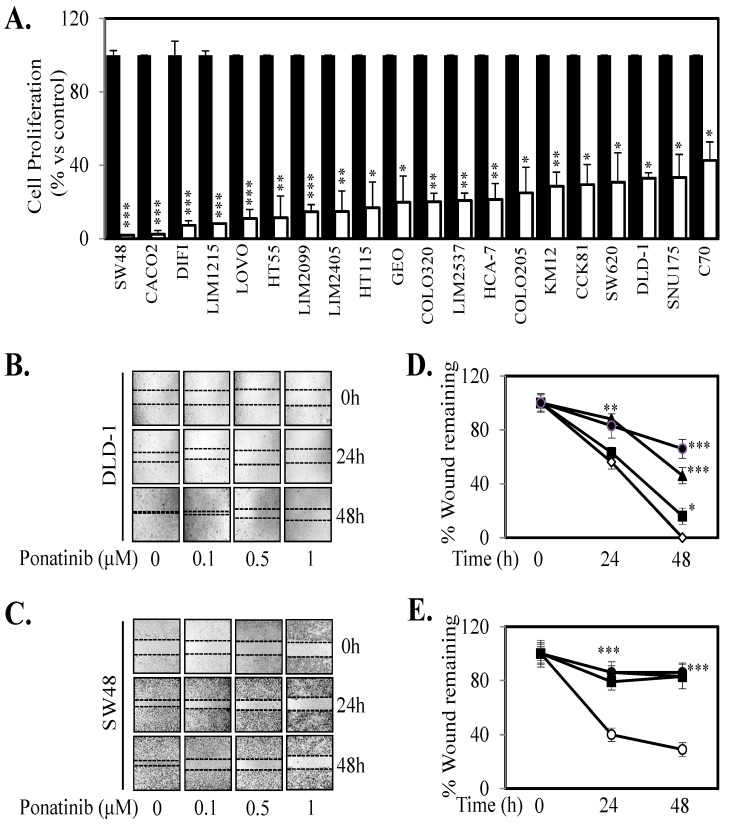
Ponatinib inhibits cell proliferation and migration. (**A**) Cells were treated with vehicle (■) or 1 µM Ponatinib (□) for 72 h. Cell viability was then determined using a commercially available Cell Titer-Glo kit and samples read on a bioluminometer. Data is expressed as % viability compared to untreated control cells ± S.D. (**B**) DLD-1 and (**C**) SW48 cells were grown to confluency, then “wounded” at time 0 h. Cells were then treated with 0, 0.1, 0.5 and 1 μM of Ponatinib for 48 h. Images of wound healing were taken at 0, 24 and 48 h post Ponatinib treatment. Graphical representation of % wound remaining relative to control treated cells at time 0 h for (**D**) DLD-1 and (**E**) SW48 cells treated with Ponatinib at 0 (○), 0.1 (■), 0.5 (▲) or 1 µM (●). Results are normalized to untreated control. Data points represent mean ± SD of at least three independent experiments, each with three experimental replicates; * *p* < 0.05; ** *p* < 0.01; *** *p* < 0.001.

**Figure 7:**
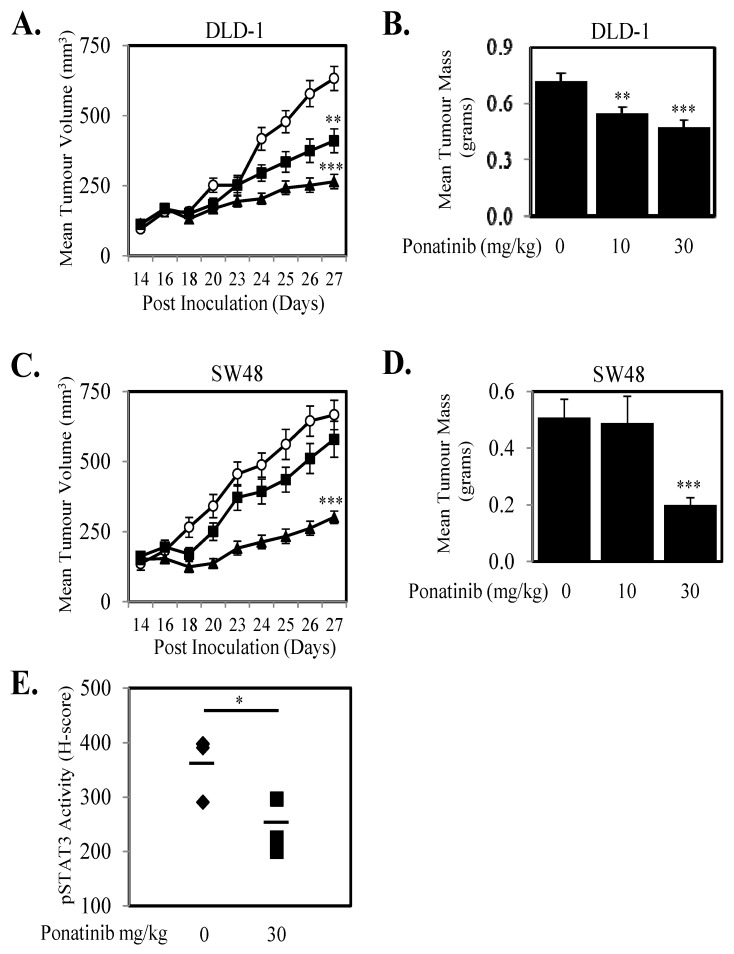
Ponatinib inhibits tumor growth in vivo. (**A**,**B**) DLD-1 and (**C**,**D**) SW48 cells were subcutaneously injected into BALB/c^nu/nu^ female mice. On day 14 when mean tumor volume had reached 100–150 mm^3^, mice were randomly separated into three groups and treated orally with daily doses of Ponatinib at 0 (○), 10 (■) or 30 mg/kg (▲) for 10 days between days 14–23 post inoculation. (**A**) DLD-1 and (**C**) SW48 data shown represents mean ± SEM (*n* = 10–12 tumors/group), and tumor mass at the end of the experiment were weighed for (**B**) DLD-1 and (**D**) SW48 and presented as mean tumor mass ± S.D. (*n* = 10–12/group) ** *p* < 0.01 and *** *p* < 0.001 relative to the vehicle treated group. (**E**) Mice bearing DLD-1 xenografts of approximately 200 mm^3^ were treated orally with Ponatinib at doses of 0 or 30 mg/kg for 2 h. Xenografts were then removed, embedded in paraffin and sections were stained for phosphorylated STAT3. H Scores were assigned based on staining intensity of at least three random fields of view from four sections from each group; * *p* < 0.05; ** *p* < 0.01; *** *p* < 0.001.

Given we demonstrate that Ponatinib could inhibit cell proliferation in seven human CRC cell lines and three primary CRC cell lines (Figure 4; Figure S4) we next tested the anti-proliferative effects of Ponatinib on a further 13 human CRC cell lines. Ponatinib facilitated greater than 50% inhibition of cell proliferation in all 20 CRC cell lines (Figure 6A) and once more displayed more consistent inhibition of proliferation of these cell lines compared to Dasatinib and Bosutinib (Table S3). Ponatinib also inhibited the “wound healing” of six CRC cell lines in a dose dependent manner (Figure 6B–E; Figure S5). Likewise, Ponatinib doses of 30 mg/kg significantly reduce tumor growth and tumor mass (Figure 7A–D) compared to tumors from control treated mice. DLD-1 xenografts also demonstrated significant reduction at the 10 mg/kg dose of Ponatinib. Importantly, Ponatinib significantly reduced STAT3 phosphorylation in tumor tissue after a single dose of 30 mg/kg compared to vehicle control (Figure 7E).

## 3. Discussion

The tumor microenvironment contains pro-oncogenic factors that support receptor activation and subsequent tumor growth and metastasis [36]. Amongst the most recognized receptors for promoting CRC progression are the EGFR, IL-6R and IL-11R systems which all activate the transcription factor, STAT3 [14,16,17]. However, STAT3 inhibitors that have entered clinical trial have produced modest results [20,21]. This is highlighted by the most recently reported trial of the STAT3 inhibitor, Napabucasin which failed to improve overall survival of patients with advanced CRC [37]. Although not a direct STAT3 inhibitor, our current study identifies an additional anti-STAT3 inhibitory property of the multi-kinase inhibitor, Ponatinib, with potent activity against STAT3 driven by the EGF/EGFR, IL-6/IL-6R and IL-11/IL-11R ligand/receptor systems.

Targeting the EGFR, which is over-expressed in up to 80% of CRC, with specific inhibitors (cetuximab, panitumumab, gefitinib and erlotinib) represents a key component of clinical management of patients with CRC [38,39]. However, considerable evidence has demonstrated that EGFR blockade, or inhibition of other receptor systems such as HER2, MET and IGF-IR leads to enhanced or re-activated STAT3 activity and subsequent continued tumor progression [24,25]. Furthermore, several reports indicate that this reactivation of STAT3 occurs through increased expression and secretion of IL-6 following EGFR blockade [24,40,41]. Our current data demonstrated that EGFR inhibitors such as gefitinib and erlotinib could not inhibit IL-6 or IL-11 mediated STAT3 activation. Thus, our current data and previous studies suggests that blockade of one receptor system that drives STAT3 activity, may not be sufficient to prevent overall STAT3 activation as other un-inhibited receptors can provide compensatory signaling to re-activate STAT3. Collectively, these studies emphasize the requirement to obstruct multiple pathways that lead to STAT3 activation, a feature unique to Ponatinib from the 1167 FDA-approved agents we screened in our study. Therefore, our findings demonstrating that Ponatinib can inhibit EGF, IL-6 and IL-11 mediated STAT3 signaling may potentially offer an improved approach to targeted therapy compared to agents that show specificity to only one pathway.

Our current data demonstrating that Ponatinib can reduce STAT3 phosphorylation, transcriptional activity, nuclear localization, dimer and tetramer formation and gene regulation indicates that Ponatinib can inhibit many critical properties of STAT3-driven tumorigenesis. Although we did not specifically show that Ponatinib could block STAT3 binding to DNA, recent evidence suggests that STAT3 dimers are required to bind DNA before forming tetramers [35]. Our data showing that Ponatinib blocked STAT3 tetramer formation indicates indirectly that Ponatinib may be able to inhibit STAT3-DNA binding. Importantly, our current data revealed that Ponatinib could induce significantly anti-tumor activity in in vivo CRC xenograft models at doses in line with that previously used in other studies in the CML and thyroid cancer settings [42,43]. As indicated by De Falco and colleagues [42] these doses are clinically relevant and thus comparable scale-up for clinical application as performed previously for CML patients would apply to CRC patients. Thus, our encouraging in vivo tumor inhibition data further advocates the repurposing of Ponatinib in the treatment management of CRC patients.

Ponatinib was structurally designed to inhibit BCR-ABL and the BCR-ABL T315I point mutation variant that confer resistance to existing tyrosine kinase inhibitors in chronic myeloid leukemia [44]. However, our data showing that Ponatinib can also inhibit STAT3 activity, correlates with other reports suggesting that Ponatinib displays broad inhibitory effects against several other targets including FLT3, c-KIT, FGFR, RET, VEGFR and PDGFR [32,42,45,46]. Collectively, these results allow for the possibility of repurposing Ponatinib for other indications including the large sub-population of patients with tumors that are dependent on STAT3 signaling. Indeed, clinical trials (NCT02272998 and NCT01813734) are ongoing based on Ponatinib’s ability to inhibit FGFR, KIT and RET [47,48]. Our current data should accelerate clinical evaluation of Ponatinib in CRC (and other tumor types) due to its anti-STAT3 activity particularly selecting patients with high STAT3 activity.

Most recently, chemical proteomics and quantitative mass spectrometry revealed that Ponatinib could bind to over 30 kinases including SRC and JAK1 [49,50]. Therefore, we compared Ponatinib’s STAT3 inhibition profile with that of five clinically approved inhibitors that have been shown to inhibit either SRC or JAK. Our data identified that Ponatinib’s broad range of anti-STAT3 inhibition is not shared by these SRC and JAK inhibitors. Ponatinib was able to inhibit STAT3 activity driven by EGF, IL-6 and IL-11 (and LIF) while Ruxolitinib, Dasatinib, Bosutinib, Ibrutinib and Tofacitinib failed to simultaneously inhibit all 3 signaling pathways. Importantly, the blockade of all three central signaling pathways that drive STAT3 activity may also lead to the delayed occurrence of acquired resistance to Ponatinib. This was evident here, as several cell lines required over four months of constant exposure to Ponatinib before we observed significant levels of acquired resistance. In contrast, we observed acquired resistance in DLD-1 cells within six weeks of continuous exposure of both Dasatinib and Bosutinib. Notably, we demonstrated that Ponatinib significantly inhibited Dasatinib and Bosutinib-refractory DLD-1 cells in vitro. This is in line with the clinical use of Ponatinib where it is used as a third line treatment option for patients that are intolerant or resistant to two or more prior TKI therapies, including Dasatinib, Nilotinib and/or Imatinib. Our current data further supports the potential clinical use of Ponatinib as an anti-STAT3 therapeutic as we speculate that patients which receive Ponatinib will require prolonged treatment regimens before acquired resistance is observed (if at all).

## 4. Materials and Methods

### 4.1. Survexpress Data Mining

SurvExpress (http://bioinformatica.mty.itesm.mx/SurvExpress) [51] was used to analyse differential gene expression of STAT3-related regulators: IL-11, IL-11R and SOCS3 comparing CRC patients with low and high expression vs. survival.

### 4.2. Antibodies and Reagents

The rabbit polyclonal antibody directed against STAT3 was obtained from Santa Cruz Biotechnology (Santa Cruz, CA, USA), while the phospho-STAT3 phospho-JAK2, JAK2 and GAPDH rabbit polyclonal antibodies were from Cell Signaling Technology (Danvers, MA, USA). IL-6, LIF and EGF were acquired from Life Technologies and IL-11 was generated in-house (Walter and Eliza Hall Institute for Medical Research, Parkville, Australia). The drug library containing 1167 FDA approved agents (including Ponatinib, Ruxolitinib, Dasatinib, Bosutinib, Ibrutinib and Tofacitinib) was obtained from SelleckChem (, Houston, TX, USA). The Luciferase Reporter Assay reagents were purchased from Promega (Madison, WI, USA). The APRE Luciferase STAT3 reporter adenovirus has been previously described [16].

### 4.3. Cell Culture

Culture of human CRC cell lines was described previously [52]. The human primary CRC cell lines CCP14, CCP19 and CPP35 were prepared from fresh biopsies as previously reported [53,54]. The DLD-1 and SW48 transfected clones were generated by transfecting cells with the IL-11Rα construct (R&D Systems, Minneapolis, MN, USA) using FuGENE HD transfection reagent (Promega, Madison, WI, USA) following the manufacturer’s instructions and selected with Geneticin (Sigma Aldrich, St. Louis, MO, USA). All cells were maintained in Dulbecco’s Modified Eagle’s Medium (Life Technologies, Carlsbad, CA, USA) contained 5% fetal bovine serum (FBS) (Life Technologies), 100 U/mL penicillin and 100 µg/mL streptomycin (Life Technologies). Cells were incubated in a humidified atmosphere of 90% air and 10% CO_2_ at 37 °C. 

### 4.4. Luciferase Assay

Cells were infected with the adenoviral STAT3 reporter (*Ad*-*APRE*-*luc*) as outlined previously [16] and allowed to adhere overnight. After 24 h, cells were stimulated with EGF (50 ng/mL), IL-6 (50 ng/mL), IL-11 (100 ng/mL) or DMSO in serum free media ± each of the 1167 inhibitors at 10 µM for our initial screen and at 1 µM for subsequent experiments where indicated further 24 h. Following another 24 h, cells were lysed and assessed for STAT3 luciferase activity with the use of the Luciferase Reporter Assay Kit (Promega) following the manufacturer’s instructions. Readings from lysed cells that were ligand stimulated (i.e., without inhibitors) were normalized to 100% and all subsequent readings were adjusted accordingly relative to ligand stimulated readings.

### 4.5. Western Blotting

Cells were lysed in a lysis buffer (50 mM Tris (pH 7.4), 150 mM NaCl, 1% Triton-X-100, 50 mM NaF, 2 mM MgCl_2_, 1 mM Na_3_VO_4_ and protease inhibitor cocktail (Roche, Basel, Switzerland)) and clarified by centrifugation (13,000× *g* for 15 min at 4 °C). Proteins were then separated by SDS-PAGE (Life Technologies), blotted onto nitrocellulose and probed with the indicated primary antibodies. The signal was visualized using an ECL chemiluminescence detection kit (GE Healthcare, Chicago, IL, USA) following incubation with appropriate secondary antibodies (Biorad Laboratories, Hercules, CA, USA).

### 4.6. Cell Viability Assays

Cells were seeded in 96-well plates and allowed to adhere overnight. Triplicate wells were then treated with varying concentrations of inhibitors where indicated for 72 h. Cells were subsequently lysed and cell viability relative to the vehicle control was determined using a commercially available Cell Titer-Glo kit (Promega) following manufacturer’s instructions. Samples were read on a bioluminometer.

### 4.7. RNA Extraction and RT-PCR

Cells were seeded in 6-well plates and allowed to adhere overnight. Following serum starvation for 24 h, cells were stimulated with ± IL-11 ± inhibitor for 8 h at 37 °C, 10% CO_2_. Total RNA was extracted with the RNeasy Mini Kit (Qiagen, Hilden, Germany) following the manufacturer’s instructions. Reverse transcription was performed using the High Capacity RNA-to-cDNA Kit (Applied Biosystems, Waltham, MA, USA) following the protocol provided from the manufacturer. Reverse Transcription-PCR was performed using the GeneAmp PCR System 2400 (Perkin Elmer, Waltham, MA, USA) under the conditions of 37 °C for 60 min and 95 °C for 5 min at a reaction volume of 20 µL. In order to quantify the transcripts of the genes of interest, real-time PCR was performed using the ViiA 7 Real-Time PCR system (Applied Biosystems) for IL-11Rα (Applied Biosystems, Hs00234415_m1), SOCS3 (Applied Biosystems, Hs02330328_s1) and GAPDH (Applied Biosystems, Hs02758991_g1). Amplified RNA samples was calculated using the 2^−ΔΔCT^ method [55]. 

### 4.8. Wound Healing Assay

Cells were seeded into 12-well plates and were cultured until 100% confluent. After which a wound was created with a p200 pipette tip. Cells were then treated with Ponatinib (0, 0.1, 0.5, 1 µM) and phase-contrast images were acquired at 0, 24 and 48 h post-scratch. An inverted microscope (IX50, Olympus, Notting Hill, Australia) and the Leica Application Suite (LAS v4.5) were used to process and capture images. ImageJ was utilized to quantify wound closure.

### 4.9. Immunohistochemistry Analysis

Paraffin-embedded tumor sections were heated to 60 °C for 1 h and deparaffinized in 100% Xylene. Slides were rehydrated in 100%, 90% and 70% ethanol followed by tap water. Antigen retrieval was performed using the BioCare Decloaking Chamber (Metagene, Redcliffe, Australia) at 110 °C for 10 min in citrate buffer pH 6.0 (Life Technologies) and cooled for 5 min in TBST. Slides were blocked in 5% goat serum followed by an endogenous peroxidases block (Envision^TM^, DAKO, North Sydney, Australia). Slides were washed in TBST followed by incubation of pSTAT3 primary antibody (1:100 dilution) overnight at 4 °C. Sections were subsequently incubated with an anti-rabbit HRP labelled polymer (Envision^TM^, DAKO) as per manufacturer’s instructions and then washed in TBST. DAB (Envision^TM^, DAKO) was then added on the sections for 5 min (RT) followed by immediate immersion in distilled water. Slides were then stained with hematoxylin for 15 s and placed in Scott’s tap water for 15 s. Following dehydration, slides were then mounted with DPX mounting media onto a coverslip and analyzed using Leica DM2000 microscope (Leica Microsystems, North Ryde, Australia). Random images were then taken and staining intensity was assessed to calculate a H-score using the following formula: 3 × percentage of cell with strong staining + 2 × percentage of cells with moderate staining + 1 × percentage of cells with weak staining. A minimum of 3 random fields were scored for each tumor section (*n* = 4/treatment group).

### 4.10. STAT3 Dimer and Tetramer Analysis

DLD-1 cells were transfected with the STAT3-EGFP construct (from Prof. Pravin Sehgal; New York Medical College, Valhalla, NY, USA). Twenty-four hours later the transfected cells were stimulated with IL-11 (100 ng/mL) with or without Ponatinib (1 µM) and immediately imaged on an Olympus FV3000 laser scanning microscope using a 60× water immersion objective (1.2 numerical aperture). The STAT3-EGFP construct was excited with a 488 nm diode pump solid state laser and its emission was detected by a GaAsP PMT between 500–600 nm. Image acquisition for number and brightness analysis of STAT3-EGFP oligomerization followed the protocol carried out previously [35,56]. Calibration of the monomeric brightness of the EGFP based construct was performed by measurement of DLD-1 cells transfected with free EGFP under identical experimental conditions. This enabled extrapolation of the expected apparent brightness of higher order EGFP oligomers (dimers and tetramers) a palette to pseudo-color brightness maps of monomeric STAT3-EGFP oligomerization upon stimulation with IL-11 ± Ponatinib. The data acquired were processed by the SimFCS software developed at the Laboratory for Fluorescence Dynamics (www.lfd.uci.edu).

### 4.11. STAT3 Nuclear Localization Analysis

DLD-1 cells were transfected with the STAT3-EGFP construct. Forty-eight hours later, the transfected cells were stimulated with IL-11 (100 ng/mL) with or without Ponatinib (1 µM) for another 1 h, before being washed twice in PBS, fixed in formaldehyde, permeabilized with PBS containing 0.2% Triton-X-100 and stained in DAPI for 2 min. Cells were then washed twice in PBS and levels of STAT3-GFP present in the cytoplasm and nucleus was determined using an Operetta High-Content Imaging System.

### 4.12. Subcutaneous Xenograft Mouse Model

DLD-1, DLD-1-IL-11R-1 and DLD-1-IL-11R-2 (2.5 × 10^6^) and SW48 SW48-IL-11R-1 and SW48-IL-11R-2 (5 × 10^6^) cells were inoculated subcutaneously into both flanks of 6–8 weeks old BALB/c nude mice (Animal Research Centre, Western Australia, Australia). Tumor volume in mm^3^ was determined as previous [57]. For experiments involving Ponatinib administration, mice were separated into three groups of five mice when tumors had reached approximately 100–150 mm^3^. Mice were subsequently treated daily by oral gavage with Ponatinib at doses of 0, 10 or 30 mg/kg for 10 days [31]. At the end of the study, tumors were collected and weighed. This research project was approved by the Animal Ethics Committee of the University of Melbourne (Ethics agreement number 1613824).

### 4.13. Statistical Analysis

All statistical analyses were performed using an unpaired, two-tail Student’s *t* test. All data sets were generated using the program GraphPad Prism6 (Prism 6.04, San Diego, CA, USA) and representing mean ± SD. Values were considered statistically significant if the *p* values was * *p* < 0.05, ** *p* < 0.01, *** *p* < 0.001. 

## 5. Conclusions

In summary, we performed a large screen of 1167 FDA-approved agents with the purpose to isolate candidate agents that could inhibit EGF, IL-6 and IL-11 mediated STAT3 activity. We successfully identified a novel mechanism for the currently FDA-approved agent Ponatinib with inhibitory properties against STAT3 activity in CRC. Moreover, we demonstrated that Ponatinib has broader and superior anti-STAT3 inhibition compared to five inhibitors with anti-SRC or anti-JAK properties and that CRC cells displayed prolonged acquired resistance to Ponatinib compared to Dasatinib and Bosutinib. Ponatinib treatment also demonstrated a significant reduction in cell viability and migration in vitro and tumor reduction in CRC xenograft mice models. Overall, our findings provide proof-of-principle evidence for the re-purposing of Ponatinib into the clinical management of CRC patients with tumors harboring elevated STAT3 activity.

## Supplementary Materials

The following are available online at http://www.mdpi.com/2072-6694/10/12/526/s1, Figure S1: Stable transfection of IL-11Rα increase STAT3 activity and SOCS3 gene expression, Figure S2: Ponatinib inhibits EGF and IL-6 mediated SOCS3 gene expression, Figure S3: Ponatinib inhibits IL-11 mediated STAT3 activity, Figure S4: Ponatinib displays broader STAT3 inhibition compared to JAK and SRC inhibitors, Figure S5: Ponatinib inhibits Cell Migration, Table S1: The effect of 1167 agents on EGF and IL-6 mediated STAT3 activity, Table S2: The effect of 50 “lead” compounds on EGF, IL-6 and IL-11 driven STAT3 transcriptional activity and phosphorylation, Table S3: The effect of Ponatinib, Dasatinib and Bosutinib on the proliferation of human colon cancer cell lines.
