# Supplementary material for: Ponatinib Inhibits Multiple Signaling Pathways Involved in STAT3 Signaling and Attenuates Colorectal Tumor Growth
Source: Cancers (Basel). 2018 Dec 19;10(12):526. doi: 10.3390/cancers10120526 (PMC6316865; doi:10.3390/cancers10120526)
Supplement: Supplementary file 1 [file cancers-10-00526-s001.pdf]

Supplementary Materials: Ponatinib Inhibits Multiple Signaling Pathways Involved in STAT3 Signaling and Attenuates Colorectal Tumor Growth

Fiona H. Tan, Tracy L. Putoczki, Jieqiong Lou, Elizabeth Hinde, Frédéric Hollande, Julie Giraud, Stanley S. Stylli, Lucia Paradiso, Hong-Jian Zhu, Oliver M. Sieber and Rodney B. Luwor

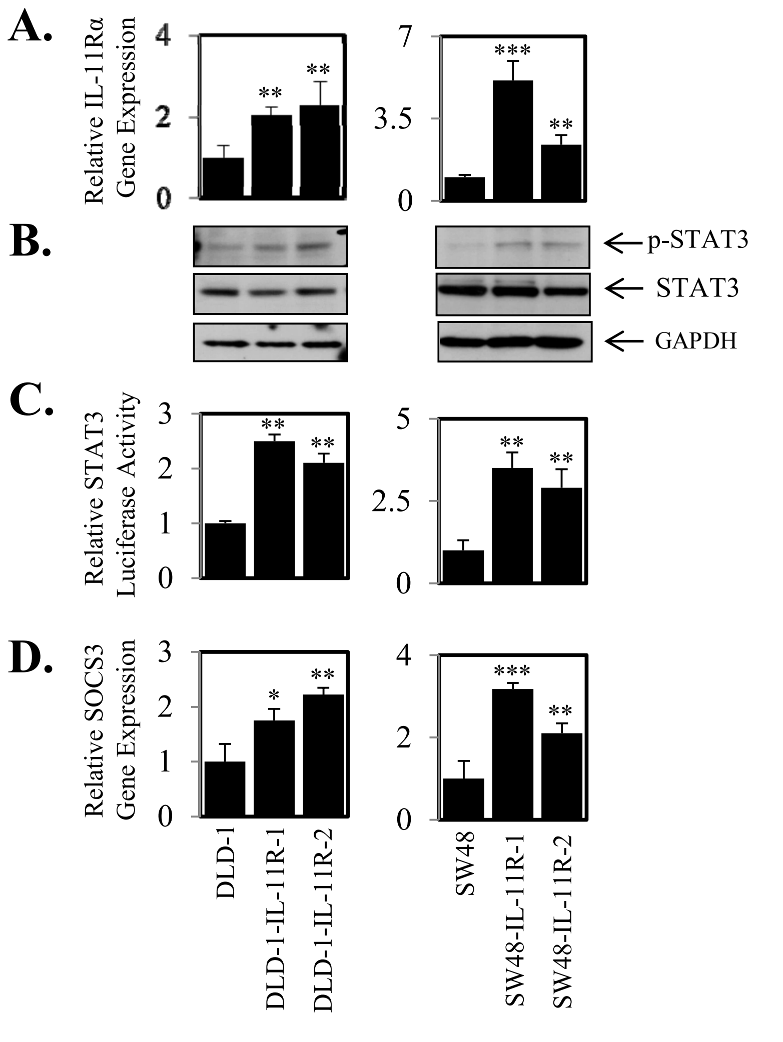

Figure S1. Stable transfection of IL-11Rα increase STAT3 activity and SOCS3 gene expression. DLD-1, DLD-1-IL-11R-1 and DLD-1-IL-11R-2 (left hand side of figure), and SW48, SW48-IL-11R-1 and SW48-IL-11R-2 (right hand side of panel) were assessed for (**A**) IL-11Rα gene expression, (**B**) phosphorylated STAT3 expression, (**C**) STAT3 luciferase transcriptional activity and (**D**) SOCS3 gene expression. All data points represent mean ± SD of at least 3 independent experiments, each with at least 3 experimental replicates; * $p < 0.05$, ** $p < 0.01$, *** $p < 0.001$ relative to control.

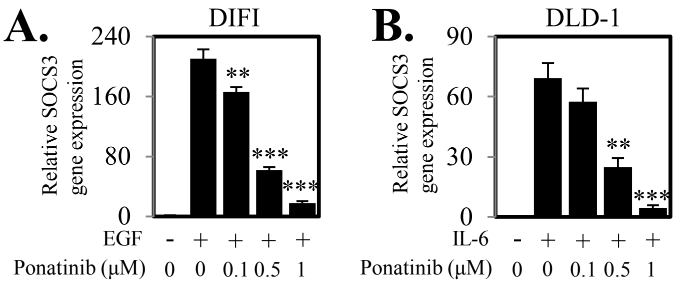

Figure S2. Ponatinib inhibits EGF and IL-6 mediated SOCS3 gene expression. (**A**) DIFI cells were stimulated with EGF ± Ponatinib and (**B**) DLD-1 were stimulated with IL-6 ± Ponatinib and then assessed for SOCS3 gene expression by qPCR; ** $p < 0.01$, *** $p < 0.001$ relative to control.

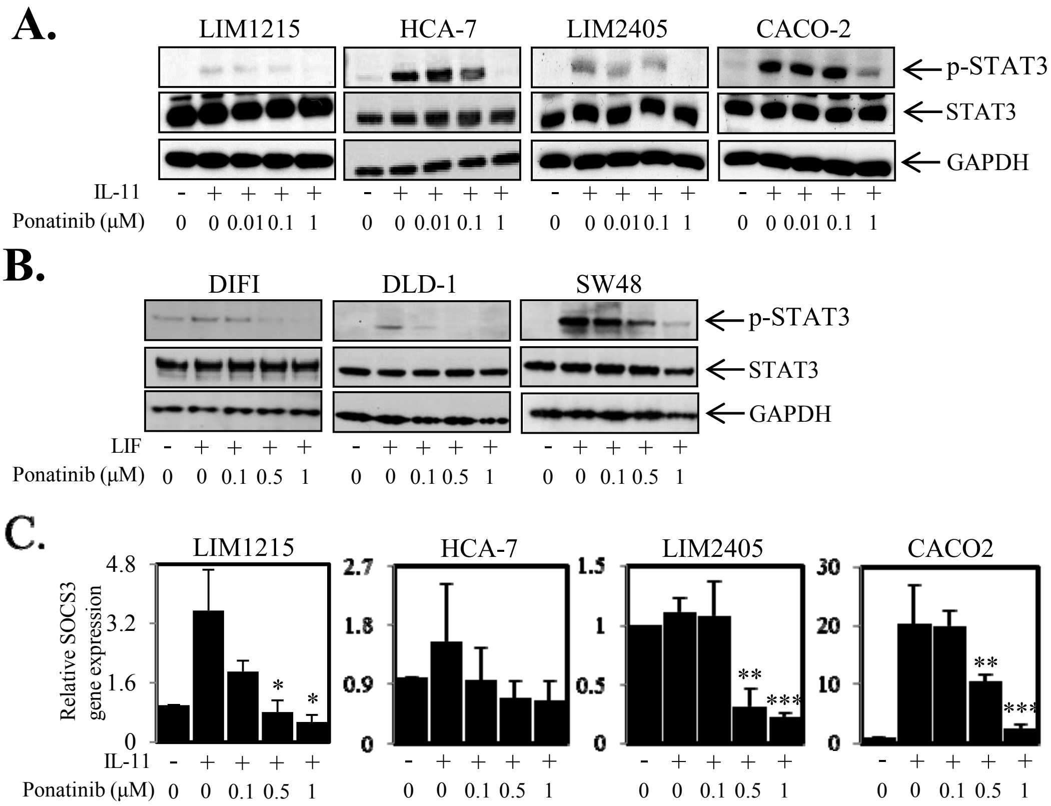

Figure S3. Ponatinib inhibits IL-11 mediated STAT3 activity. (**A**) Cells were stimulated with IL-11 ± Ponatinib and then assessed for Phospho-STAT3, STAT3 and GAPDH expression by western blot. (**B**) Cells were treated with IL-11 ± Ponatinib and then assessed for SOCS3 gene expression by qPCR; * $p < 0.05$, ** $p < 0.01$, *** $p < 0.001$ relative to control. (**C**) Cells were treated with LIF ± Ponatinib, and then assessed for Phospho-STAT3, STAT3 and GAPDH expression by western blot.

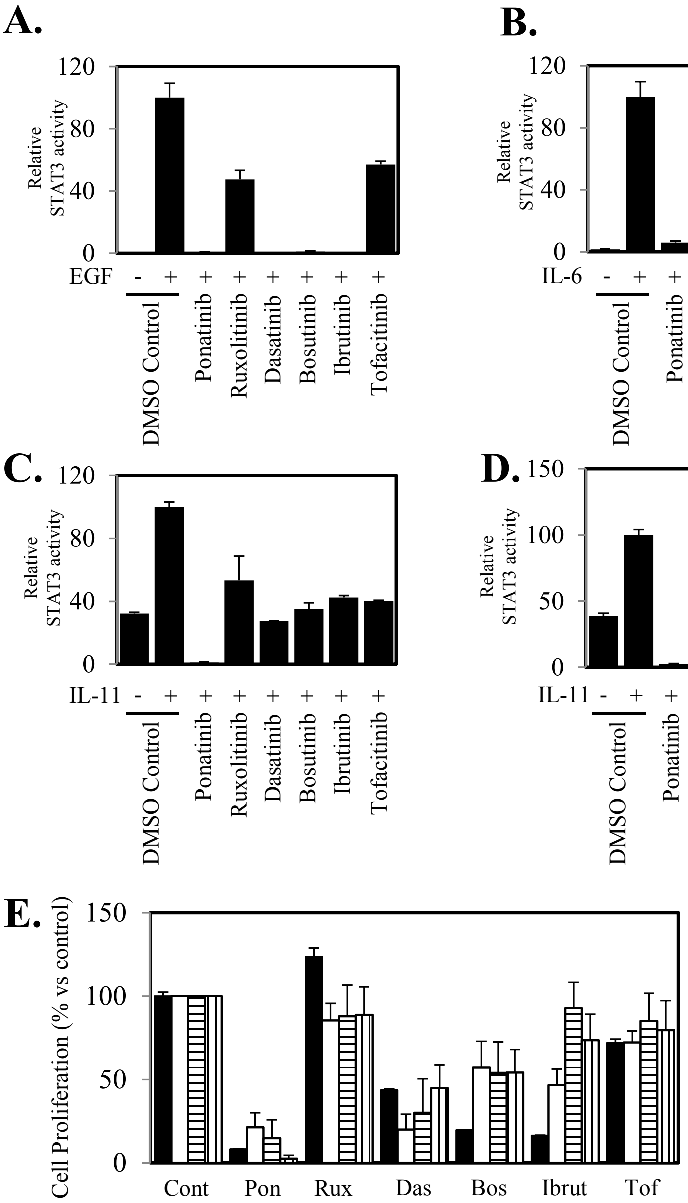

Figure S4. Ponatinib displays broader STAT3 inhibition compared to JAK and SRC inhibitors. Cells were infected with Ad-APRE-luc adenovirus then stimulated with (**A**) EGF (DIFI), (**B**) IL-6 (DLD-1), (**C**) IL-11 (DLD-1), (**D**) IL-11 (SW48) ± 1 μM Ponatinib, Ruxolitinib, Dasatinib, Bosutinib, Ibrutinib or Tofacitinib for 24 h. STAT3 reporter activity was then determined as outlined in Materials and Methods. Data represents percentage luciferase activity relative to ligand stimulated DMSO control, mean ± SD of 3 independent experiments. (**E**) LIM1215 (■), HCA-7 ( ), LIM2405 (horizontal lines) and CACO2 (vertical lines) were treated with ± Ponatinib (Pon), Ruxolitinib (Rux), Dasatinib (Das), Bosutinib (Bos), Ibrutinib (Ibrut) or Tofacitinib (Tof) for 72 h. Cell viability was then determined using a commercially available Cell Titer-Glo kit and samples read on a bioluminometer. Data is expressed as % viability compared to untreated control cells ± S.D.

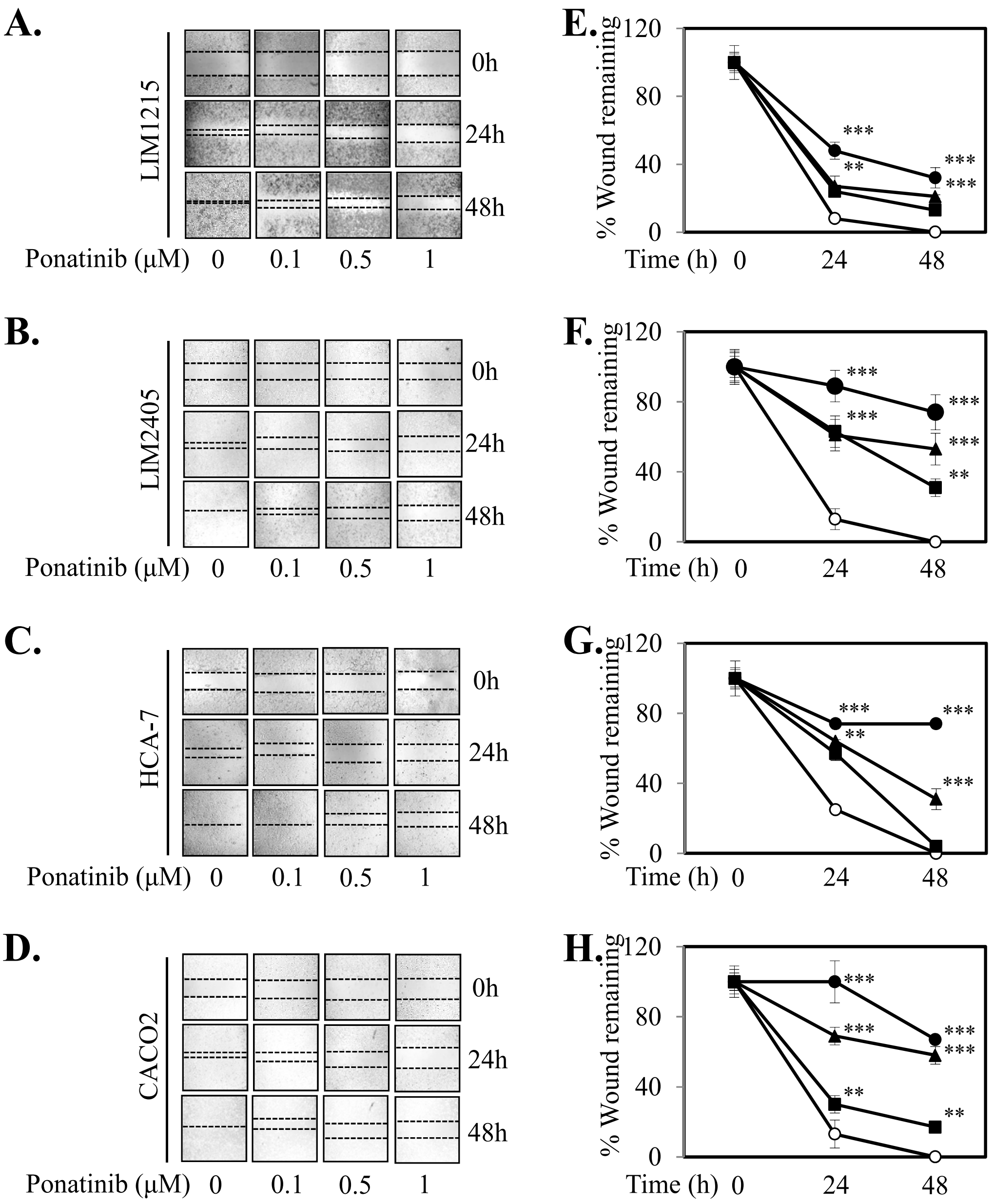

Figure S5. Ponatinib inhibits Cell Migration. (**A**) LIM1215, (**B**) LIM2405, (**C**) HCA-7 and (**D**) CACO2 cells were grown to confluency, then "wounded" at time 0 h. Cells were then treated with 0. 0.1, 0.5 and 1 μM of Ponatinib for 48 h. Images of wound healing were taken at 0, 24 and 48 h post Ponatinib treatment. Graphical representation of % wound remaining relative to control treated cells at time 0 h for (**E**) LIM1215, (**F**) LIM2405, (**G**) HCA-7 and (**H**) CACO2 cells treated with Ponatinib at 0 (○), 0.1 (■), 0.5 (▲) or 1 μM (•). Results are normalized to untreated control. Data points represent mean ± SD of at least 3 independent experiments, each with 3 experimental replicates; ** $p < 0.01$; *** $p < 0.001$.

Table S1. The effect of 1167 agents on EGF and IL-6 mediated STAT3 activity.

STAT3 Activity (%)	Profile of Agents Tested Against EGF Mediated STAT3 Activity (%)	Profile of Agents Tested Against IL-6 Mediated STAT3 Activity (%)
≥100	626 (53)	437 (37)
75–<100	382 (33)	527 (45)
50–<75	70 (6)	111(10)
25–<50	36 (3)	42 (4)
0–<25	53 (5)	50 (4)
**Total**	**1167 (100)**	**1167 (100)**

Table S2. The effect of 50 "lead" compounds on EGF, IL-6 and IL-11 driven STAT3 transcriptional activity and phosphorylation.

**Agent**	**EGF Driven STAT3 Transcriptional Activity (% ± S.D.)**	**IL-6 Driven STAT3 Transcriptional Activity (% ± S.D.)**	**EGF Driven STAT3 Phosphorylation (% vs. Control)**	**IL-6 Driven STAT3 Phosphorylation (% vs. Control)**		**IL-11 Driven STAT3 Transcriptional Activity (% ± S.D.)**		
Cell line (Treatment Dose)	DIFI (10 μM)	DLD-1 (10 μM)	DIFI (1 μM)	DLD-1 (1 μM)	DLD-1 (10 μM)	DLD-1 (1 μM)	SW48 (1 μM)	LIM1215 (1 μM)
DMSO Control	100 ± 3.8	100 ± 4.9	100	100	100	100 ± 3.2	100 ± 4.1	100 ± 5.3
Alexidine HCl	0.1 ± 0.0	0.0 ± 0.0	28.9	47.5	0.3	10.4 ± 0.3	19.3 ± 0.1	73.8 ± 0.1
Azacitidine	20.8 ± 3.2	35.1 ± 2.4	99.5	40.3	38.4	142.8 ± 1.4	126.3 ± 1.6	83.6 ± 4.0
Azelnidipine	5.5 ± 1.3	19.5 ± 3.2	99.9	98.7	97	80.2 ± 0.2	47.8 ± 4.3	171.9 ± 1.6
Benzbromarone	14.1 ± 3.7	4.7 ± 1.2	52.9	66.8	65.8	31.9 ± 5.0	89.6 ± 1.1	85.9 ± 11.5
Bexarotene	22.2 ± 5.7	23.8 ± 3.8	97.7	97	25.1	62.0 ± 1.0	74.9 ± 3.7	80.2 ± 4.6
Bortezomib	8.3 ± 1.2	3.2 ± 3.6	101.6	75.8	75.4	13.8 ± 0.8	16.3 ± 0.5	29.2 ± 3.7
Bosutinib	3.4 ± 1.4	40.5 ± 5.4	0.1	99.4	94	35.2 ± 3.9	125.7 ± 3.3	13.6 ± 0.7
Broxyquinoline	17.8 ± 4.6	23.4 ± 3.5	69.4	79.7	69.6	40.4 ± 0.6	29.5 ± 0.4	115.1 ± 9.1
Carfilzomib	2.9 ± 0.7	7.1 ± 2.4	98.3	99.5	99	15.1 ± 0.1	13.9 ± 1.8	39.4 ± 1.5
Cetrimonium Bromide	0.1 ± 0.0	0.0 ± 0.0	60.4	70.7	5	32.5 ± 12.2	32.7 ± 1.2	49.0 ± 1.0
Cetylpyridinium Chloride	0.1 ± 0.0	0.0 ± 0.0	52.8	69.8	0.5	14.5 ± 0.2	17.0 ± 0.4	26.4 ± 1.7
Chlorquinaldol	4.3 ± 1.1	17.2 ± 4.5	82.2	85.4	44.8	5.0 ± 0.6	4.0 ± 0.3	103.3 ± 1.4
Closantel Sodium	12.4 ± 2.9	0.2 ± 0.0	54.4	30.9	3	4.4 ± 0.2	101.0 ± 8.7	110.1 ± 0.7
Closantel	0.1 ± 0.0	0 ± 0.0	37.6	21.1	14.8	13.0 ± 0.2	118.1 ± 1.4	106.9 ± 3.7
Cyclosporine	36.6 ± 3.2	30.0 ± 4.6	99	76.1	35.5	26.5 ± 0.6	39.4 ± 0.7	23.4 ± 1.4
Daunorubicin HCl	0.1 ± 0.0	0.4 ± 0.1	100.2	98.8	99.9	35.6 ± 7.6	15.2 ± 0.5	21.0 ± 10.9
Dasatinib	0.1 ± 0.0	44.3 ± 8.8	22.1	95.5	94.3	27.4 ± 0.3	102.3 ± 3.8	33.3 ± 0.9
Diethylstilbestrol	43.2 ± 4.3	22.2 ± 4.7	99	31.8	16.1	81.9 ± 0.7	139.6 ± 1.5	110.2 ± 3.7
Domiphen Bromide	0.1 ± 0.0	0.0 ± 0.0	88.9	100.4	88	57.0 ± 1.1	23.6 ± 1.6	80.7 ± 1.3
Doxorubicin	2.0 ± 1.5	31.8 ± 5.6	98.3	44.8	31.1	81.1 ± 4.1	26.7 ± 0.5	137.4 ± 0.8
Dronedarone HCl	19.1 ± 3.4	38.3 ± 3.7	91.3	98.8	26.4	71.1 ± 0.4	89.2 ± 2.0	74.9 ± 2.7
Elvitegravir	30.3 ± 2.4	21.7 ± 3.7	37.1	31.5	28.2	88.4 ± 1.4	92.7 ± 1.8	115.5 ± 12.6
Emetine	3.6 ± 0.2	0.0 ± 0.0	93.8	69.7	68.5	1.4 ± 0.1	5.5 ± 0.4	6.2 ± 0.2
Epirubicin HCl	47.5 ± 3.7	17.1 ± 1.7	98.6	83.2	77.3	130.8 ± 4.4	25.4 ± 0.3	167.6 ± 0.5
Erlotinib HCl	0.1 ± 0.0	40.7 ± 4.2	0.6	98.2	81.1	38.5 ± 0.9	65.8 ± 0.3	91.8 ± 0.5
Flunarizine 2HCl	30.0 ± 2.8	36.5 ± 4.3	49.1	28.8	26.8	53.9 ± 1.7	38.3 ± 1.0	137.6 ± 3.1
Fludarabine Phosphate	39.9 ± 2.6	4.5 ± 1.5	99.5	54.3	34.3	24.3 ± 1.3	3.0 ± 0.2	16.0 ± 1.9
Irinotecan	38.9 ± 1.4	37.9 ± 2.4	104.5	95	94.3	105.3 ± 15.6	55.6 ± 0.5	185.4 ± 5.1
Ivermectin	2.0 ± 0.2	0.7 ± 0.1	99.1	47.2	17.1	13.3 ± 0.4	53.0 ± 3.6	136.1 ± 1.6

Miconazole nitrate	37.5 ± 3.9	16.1 ± 2.6	84.4	97.9	21.4	63.5 ± 1.0	77.5 ± 0.8	93.4 ± 1.2
Mitoxantrone HCl	14.0 ± 2.6	16.5 ± 3.9	100.2	97.6	98.1	148.5 ± 3.0	27.8 ± 0.9	341.7 ± 5.4
Montelukast Sodium	6.3 ± 1.4	2.0 ± 0.3	99.6	66.3	59.2	59.6 ± 11.4	107.2 ± 3.2	104.7 ± 4.7
Mycophenolate mofetil	36.1 ± 5.4	21.0 ± 3.6	51	82.2	78.2	16.0 ± 0.3	78.2 ± 1.1	193.2 ± 1.2
Mycophenolic	23.0 ± 5.4	9.9 ± 2.1	98.9	99.1	99.7	45.7 ± 0.4	67.3 ± 3.0	171.8 ± 6.0
Niclosamide	25.3 ± 2.4	4.4 ± 1.2	86.4	96.4	97.2	0.4 ± 0.1	5.6 ± 0.2	17.1 ± 0.4
OSI-420	0.2 ± 0.0	22.1 ± 2.5	0.1	98.5	96.9	64.6 ± 2.2	87.2 ± 0.7	129.0 ± 8.6
Ouabain	1.0 ± 0.2	1.7 ± 0.8	91.9	82.4	75.8	12.4 ± 0.8	2.4 ± 0.2	9.8 ± 1.4
Penfluridol	48.9 ± 4.7	49.2 ± 4.1	63.2	84.8	35.1	66.9 ± 1.5	42.0 ± 0.5	85.3 ± 1.5
**Ponatinib**	3.1 ± 0.2	3.8 ± 0.7	18.9	0.2	0.2	1.1 ± 0.1	2.6 ± 0.2	7.9 ± 0.9
Pyrithione zinc	0.1 ± 0.1	0 ± 0.0	44.1	56.9	5.9	0.4 ± 0.2	4.9 ± 2.5	32.4 ± 0.5
Regorafenib	37.7 ± 6.9	41.6 ± 3.2	99.3	104.6	30.8	13.3 ± 0.5	14.3 ± 2.1	34.6 ± 1.6
Rimonabant	47.6 ± 2.5	6.8 ± 1.3	100.4	99.3	99.2	14.9 ± 0.2	14.8 ± 0.8	79.3 ± 3.2
Sertaconazole nitrate	39.4 ± 4.7	9.7 ± 2.5	78.9	87.3	44	22.9 ± 0.6	110.2 ± 6.8	107.2 ± 4.1
Sorafenib	12.6 ± 1.4	32.8 ± 1.8	95.6	96.1	12.2	43.5 ± 1.8	68.0 ± 1.1	65.0 ± 1.8
Sunitinib Malate	48.1 ± 3.4	28.3 ± 1.6	96.1	90	45.8	79.4 ± 7.0	108.1 ± 1.8	106.1 ± 0.6
Thonzonium Bromide	6.3 ± 2.3	24.1 ± 3.4	84.3	71.9	73.4	61.8 ± 1.7	69.4 ± 3.2	41.9 ± 1.3
Tiratricol	33.9 ± 3.2	5.3 ± 1.6	92.8	102.6	81	79.4 ± 0.9	90.7 ± 0.8	109.7 ± 6.3
Topotecan HCl	10.6 ± 1.9	1 ± 0.0	91	62.8	50.6	47.1 ± 1.2	31.5 ± 0.9	80.3 ± 0.6
Triclabendazole	34.1 ± 2.7	13.1 ± 3.6	54.7	76.1	42.2	44.8 ± 0.2	67.1 ± 0.7	142.9 ± 3.9
Vandetanib	0.0 ± 0.0	47.6 ± 1.8	0.3	100.6	0.2	51.8 ± 1.9	63.4 ± 0.6	80.6 ± 0.7
Vorinostat	10.2 ± 0.9	22.6 ± 2.5	101.8	96.3	93.9	706.9 ± 5.4	348.5 ± 4.3	770.3 ± 4.4

Table S3. The effect of Ponatinib, Dasatinib and Bosutinib on the proliferation of human colon cancer cell lines.

Cell Line	Reduction vs DMSO Control Treated Cells (% ± S.D.)		
	Ponatinib	Dasatinib	Bosutinib
SW48	2.0 ± 0.3	20.7 ± 0.8	65.5 ± 1.9
CACO2	2.4 ± 2.0	44.7 ± 14.0	54.2 ± 13.7
DIFI	7.2 ± 2.6	14.5 ± 3.8	3.5 ± 0.8
LIM1215	8.2 ± 0.3	43.5 ± 0.8	19.6 ± 0.3
LOVO	11.0 ± 4.9	28.6 ± 16.1	44.1 ± 16.7
HT55	11.4 ± 11.9	46.6 ± 20.5	30.1 ± 18.7
LIM2099	14.6 ± 3.8	34.0 ± 11.7	60.0 ± 20.3
LIM2405	14.8 ± 11.1	30.0 ± 20.6	54.0 ± 18.5
HT115	16.8 ± 14.2	42.7 ± 16.0	65.1 ± 27.7
GEO	19.9 ± 14.3	28.0 ± 18.3	26.0 ± 15.9
COLO320	20.1 ± 4.6	69.3 ± 17.0	79.8 ± 19.8
LIM2537	20.8 ± 4.0	64.3 ± 15.1	63.3 ± 16.1
HCA-7	21.3 ± 8.7	19.9 ± 9.4	57.1 ± 15.6
COLO205	25.0 ± 13.9	36.0 ± 22.2	73.1 ± 22.9
KM12	28.6 ± 7.5	78.2 ± 14.0	45.4 ± 11.7
CCK81	29.4 ± 10.9	27.0 ± 9.3	40.7 ± 14.1
SW620	30.7 ± 16.0	68.0 ± 22.6	114.3 ± 20.8
DLD-1	33.0 ± 2.9	51.0 ± 7.6	53.9 ± 6.8
SNU175	33.2 ± 12.6	53.1 ± 18.5	44.8 ± 18.8
C70	42.7 ± 10.0	41.5 ± 23.5	42.5 ± 22.7

© 2018 by the authors. Licensee MDPI, Basel, Switzerland. This article is an open access article distributed under the terms and conditions of the Creative Commons Attribution (CC BY) license (http://creativecommons.org/licenses/by/4.0/).
